# Cognitive and Behavioural Profile of SOD1-ALS Across the ALS-FTD Spectrum: A Systematic Review

**DOI:** 10.3390/ijms27177958

**Published:** 2026-09-07

**Authors:** Francesco Ginanni, Francesco Nicoletti, Caterina Meoni, Lucrezia Becattini, Michelangelo Mancuso, Cecilia Carlesi, Francesca Bianchi

**Affiliations:** 1 Department of Clinical and Experimental Medicine, Neurological Institute, University of Pisa, 56126 Pisa, Italy; francesco.ginanni99@gmail.com (F.G.); francesco.nicoletti99@gmail.com (F.N.); cmeoni97@gmail.com (C.M.); lu.becattini@gmail.com (L.B.); 2 Neurology Unit, Azienda Ospedaliero Universitaria Pisana, 56126 Pisa, Italy; cecilia.carlesi@gmail.com (C.C.); francicr86@gmail.com (F.B.); 3 CTMM—Interdepartmental Research Centre for Translational Medicine in Neuromuscular and Mitochondrial Diseases, University of Pisa, 56126 Pisa, Italy

**Keywords:** amyotrophic lateral sclerosis (ALS), SOD1, frontotemporal dementia (FTD), cognitive impairment, behavioural impairment, systematic review, neuropsychology

## Abstract

Amyotrophic Lateral Sclerosis (ALS) is increasingly recognized as a multisystem disorder. However, *SOD1*-associated ALS has traditionally been regarded as an exclusively motor phenotype. This systematic review challenges that paradigm by characterizing the cognitive and behavioural profiles of *SOD1* mutation-related ALS. Following PRISMA guidelines, a systematic PubMed search identified 17 eligible studies. Across the included studies, encompassing 222 patients with *SOD1* variants, three of the analyzed subjects fulfilled the diagnostic criteria for frontotemporal dementia (FTD), and a larger proportion exhibited multidomain cognitive deficits, including executive dysfunction, language impairment, and working memory decline. Behavioural disturbances, such as apathy, emotional lability, and mental rigidity, were also prominent. The historical assumption that *SOD1* mutations completely spare cognitive networks likely reflects an under-recognition of subtle manifestations, compounded by the limited sensitivity of earlier screening tools. These findings indicate that cognitive and behavioural involvement can occur in *SOD1*-ALS, highlighting a highly heterogeneous phenotype. Encompassing varying degrees of severity—from subtle differences in neuropsychological test scores to formal FTD diagnoses—the current evidence points towards possible variant-specific phenotypes rather than a uniform cognitive syndrome. Routine multidomain cognitive screening is crucial in this population. Properly characterizing these non-motor features allows for more accurate disease staging, better monitoring of clinical progression, and the implementation of truly personalized care strategies.

## 1. Introduction

Amyotrophic Lateral Sclerosis (ALS) is a neurodegenerative disorder long considered to purely involve the motor system. In recent years, a deeper understanding of the disease has shed light on the multifaceted nature of this disorder, which is no longer considered purely motor. Indeed, several non-motor systems may also be affected, similarly to what has been observed in other neurodegenerative disorders [1,2,3].

ALS is characterized by great heterogeneity from both clinical and pathogenetic perspectives. While motor involvement—characterized by motor neuron degeneration—remains the hallmark of the disease, the importance of cognitive involvement has gradually emerged over the last twenty years of research [4,5]. Approximately half of all ALS patients develop cognitive-behavioural manifestations during the disease course. In most cases, this involvement manifests as executive dysfunction and mild memory decline. However, in a minority of cases, the disorder may progress to more severe cognitive impairment, taking on the characteristics of overt dementia [6].

ALS is now considered a multisystem condition that shares a clinical, pathological, and genetic continuum with frontotemporal dementia (FTD). Between the two opposite poles—purely motor ALS and purely FTD—several shades of motor and cognitive involvement have been described by Strong et al. [2,7]. The Strong criteria, originally introduced in 2009 and later revised, formally classified several cognitive and behavioural profiles associated with ALS, distinguishing between ALS with cognitive impairment (ALSci) and ALS with behavioural impairment (ALSbi). These criteria facilitated the formal characterization of those alterations observed for a long time in motor neuron disease.

In the past twenty years, studies conducted on the neuropsychological profile of ALS patients have highlighted a predominant impairment in free recall, executive functions, and naming, alongside relatively preserved attention, psychomotor speed, and visuospatial function [8].

However, the considerable clinical heterogeneity of ALS from a neuropsychological perspective highlighted the need for more rigorous diagnostic standardization. In 2017, a study coordinated by the University of Edinburgh and the University of Pennsylvania formalized a revision of the diagnostic criteria for the frontotemporal spectrum of disorders associated with ALS, defining this complex spectrum of clinical phenotypes as Amyotrophic Lateral Sclerosis-Frontotemporal Spectrum Disorder (ALS-FTSD) [2].

This revision enabled a more accurate and precise assessment of cognitive-behavioural impairment in these patients by focusing on the specific domains affected by the pathology (executive functions, verbal fluency, and social cognition), identifying four clinical categories: ALSci, ALSbi, ALScbi, and ALS-FTD. The revision of the Strong criteria allowed for the standardization of neuropsychological profiling in patients with motor neuron disease, thereby enabling a more precise definition of the phenotype [2].

Within the complex clinical continuum, genetics plays a central role in defining the broad spectrum of phenotypic presentations [9]. In 85–90% of cases, the disease is considered sporadic (SALS), while the remaining 10–15% consist of familial cases (FALS), usually inherited in an autosomal dominant manner [9].

Patients with familial ALS frequently exhibit a pathogenic mutation in one of the major ALS-associated genes: *C9orf72*, *SOD1*, *TARDBP*, or *FUS*. In addition to these four genes, at least 30 other genes involved in the disease’s pathogenesis have been described [10], bringing the total proportion of identifiable pathogenic variants to approximately 25% of cases [11].

The familial ALS cases related to pathogenic variants in the four main genes (*C9orf72*, *SOD1*, *TARDBP*, and *FUS*) are associated with a specific disease phenotype, clinical course, and neuropsychological profile [12]. Indeed, although variables like age at disease onset and gender contribute to defining the clinical phenotype, the genetic profile represents one of the most significant determinants of the disease’s heterogeneity [13].

Among the several pathogenetic mutations in ALS, the expansion of *C9orf72* is widely recognized as a major genetic substrate shared by several neurodegenerative disorders, including Parkinson’s disease and Alzheimer’s disease [14]. The pathological expansion of *C9orf72* is the most frequent genetic cause of both fALS and FTD, and patients carrying this mutation exhibit a higher risk of developing the ALS-FTD profile [15].

Patients carrying the *C9orf72* repeat expansion exhibit a distinctive disease phenotype characterized by earlier disease onset, bulbar involvement from the early stages of the disease, a higher prevalence of cognitive and behavioural impairment, a family history of autosomal dominant neurodegenerative diseases, and reduced survival [16]. From a cognitive perspective, these patients are more likely to develop cognitive deficits primarily affecting executive functions and verbal memory. Regarding the latter, verbal memory appeared to be affected even in those patients who did not reach the diagnostic criteria for cognitive impairment [16,17]. When tested with the Edinburgh Cognitive and Behavioural ALS Screen (ECAS) scale—a neuropsychological test often used as a screening test of cognitive status in ALS—an inverse correlation has been described between the length of the *C9orf72* hexanucleotide repeat expansion and performance on the ECAS test [16,17].

Unlike the *C9orf72* gene mutation—widely recognized as a major genetic determinant of cognitive impairment in ALS—the contribution of other ALS-associated genes, including *SOD1*, remains controversial [18]. Indeed, until relatively recently, the characterization of the cognitive phenotype associated with *SOD1* mutations relied primarily on individual case reports and small case series [19]. Consequently, the relationship between *SOD1* mutations and cognitive dysfunction remains incompletely understood.

The following discussion focuses on several controversial aspects of the effects of *SOD1* gene mutations on cognitive function that continue to be actively debated in the scientific literature. Since 1993, more than 200 pathogenic variants of the *SOD1* gene have been identified [20]. The majority are autosomal dominant heterozygous mutations, with a toxic gain-of-function pathogenic effect [21].

Historically, ALS phenotypes associated with *SOD1* mutations have been considered exclusively motor. However, in recent years, this axiom has been increasingly challenged [18,19]. A 2005 Italian multicenter study provided some of the first evidence of a possible cognitive-behavioural involvement in ALS associated with *SOD1* gene mutations. In this study, among the 264 ALS patients enrolled, including 39 patients with familial ALS, 7 patients (17.9%) carried a *SOD1* gene mutation. Among the various mutations identified in this gene, the p.Gly41Ser mutation in exon 5 was the most frequent, being detected in four patients. Two of these patients—who belonged to the same family—exhibited behavioural abnormalities from disease onset, characterized by severe social conduct disturbances and apathy. However, the study did not provide an in-depth assessment of the patients’ cognitive profiles, focusing instead on their behavioural profiles.

With the growing evidence of possible cognitive-behavioural involvement in patients with *SOD1* mutations, Wicks et al. [22] conducted a study in 2009 involving 58 subjects divided into four groups: (i) patients with sporadic ALS; (ii) patients with familial ALS not associated with *SOD1* mutation; (iii) *SOD1*-related ALS patients, and (iv) healthy controls. The study revealed that, pertaining to cognition, patients with the *SOD1* mutation showed generally preserved cognitive functions, while, pertaining to behaviour, ALS patients—including those carrying *SOD1* mutations—exhibited higher levels of apathy and emotional lability compared to healthy controls. This study further demonstrated that patients with *SOD1* mutations may exhibit behavioural changes, providing evidence that behavioural impairment can occur even in *SOD1*-associated ALS, which was historically considered to affect exclusively the motor system [22].

In another study conducted by Marjanović et al. [23] on a cohort of 175 ALS patients (23 familial and 152 sporadic cases), pathogenic *SOD1* mutations were identified in 21.7% of familial cases and 1.3% of sporadic cases. Patients with *SOD1*-ALS showed better performance in tests exploring general intelligence, verbal and visuospatial memory, language, and executive functions compared to *SOD1*-negative ALS patients. However, their scores on the Stroop Colour-Word Test were pathological, suggesting a selective, mild impairment of inhibitory control processes [23].

Recent research has revealed a more heterogeneous neuropsychological scenario, demonstrating that *SOD1* patients may also display cognitive impairment. A study by Calvo et al. (2024) [18] involving a large cohort of ALS patients enrolled at the CRESLA Center demonstrated that twelve (42.9%) of the *SOD1*-mutated patients exhibited cognitive and/or behavioural impairment at the time of diagnosis. However, none of these patients met the criteria for ALS-FTD [18]. Compared to healthy controls, ALS patients with *SOD1* mutations showed multidomain deficits, primarily affecting executive functions, working memory, non-verbal intelligence, and social cognition. Moreover, when compared to sporadic ALS, *SOD1*-mutated patients showed a more frequent impairment in social cognition and language domains, while executive functions were less affected [18].

Interestingly, a higher prevalence of anxiety and depression was observed in *SOD1* patients, confirming that behavioural changes are often detected in these patients. Similarly to what happens in other types of neurodegenerative disorders, older age at onset and low educational attainment were identified as risk factors for the development of cognitive impairment—suggesting that this process follows the same deleterious and protective mechanisms observed in dementia [18].

A single-centre study published in 2022 by Dalla Bella et al. [24] that compared the cognitive and behavioural profiles of patients with sporadic ALS with those carrying *SOD1* mutations documented that approximately 60% of the entire patient cohort exhibited cognitive and/or behavioural impairment. However, while cognitive abnormalities were more frequent in patients with sporadic ALS, those with *SOD1* mutations primarily displayed behavioural alterations—mainly characterized by irritability and mental rigidity—especially in those mutations involving exon 5. These alterations were independent of age and disease stage [24]. A common limitation of most studies published so far is that patients with *SOD1* mutations have been considered as a single clinical category, without distinguishing between the gene’s numerous pathogenic variants [24].

This limitation was addressed by Winroth et al. (2024) [25] through a study that involved a cohort of 48 ALS patients and 37 healthy controls. The study analyzed participants’ neuropsychological profiles, particularly focusing on patients homozygous for the p.Asp91Ala (D91A) mutation in *SOD1*. This mutation shows a high prevalence in Scandinavia and is characterized by a very slow progression of motor symptoms. Overall, the study revealed a higher prevalence of cognitive impairment in *SOD1*-ALS patients compared to healthy subjects, though the cognitive decline was milder and distinct from that registered in patients with the *C9orf72* mutation. ALS patients showed deficits in working memory, regardless of *SOD1* mutation status. Regarding visuospatial performance, patients with *SOD1* mutations performed better than both patients with sporadic ALS and *C9orf72* mutations, while language was generally preserved [25].

Regarding the cognitive profile of patients carrying the p.Asp91Ala mutation, these individuals exhibited cognitive performance that was comparable to or exceeded that of patients harbouring other *SOD1* mutations across most cognitive domains. The only exception was represented by a more pronounced impairment in complex working memory. Overall, these findings suggested that the p.Asp91Ala variant may be associated with a relatively preserved cognitive phenotype [25].

Conversely, a global meta-analysis examining the neuropsychological spectrum across distinct ALS genotypes [26] demonstrated that the *SOD1* gene exerts a negligible effect on the overall cognitive profile. When compared directly to the severe executive and behavioural impairment typically driven by *C9orf72* repeat expansions or *PGRN* variants—which are strongly linked to frontotemporal lobar degeneration—*SOD1* mutations demonstrated a remarkably preserved cognitive status. Consequently, the authors concluded that while subtle domain-specific deficits may occasionally occur, the overall burden of cognitive dysfunction attributable to *SOD1* variants remains very modest on a population level [26].

Overall, these studies provide converging evidence that *SOD1*-related ALS should no longer be regarded as a disorder exclusively affecting the motor system. Moreover, the marked clinical and molecular heterogeneity associated with *SOD1* mutations suggests that this genetic form of ALS is unlikely to be characterized by a single, uniform neuropsychological phenotype. A single-centre cohort study employing highly sensitive, standardized test batteries by Calvo et al. (2024) [18] highlighted a higher prevalence (exceeding 40%) of cognitive-behavioural impairment in patients harbouring *SOD1* mutations, mainly characterized by selective impairments in social cognition and language.

However, these divergent epidemiological findings likely reflect cohort-specific clinical phenotypes rather than a uniform disease spectrum. The neuropsychological profile of patients with *SOD1*-ALS is heavily influenced by geographic selection biases and founder effects, as specific variants—such as the Scandinavian p.Asp91Ala mutation or exon 5 variants in Mediterranean cohorts—predominate in distinct regional populations.

The discrepancies between existing literature and recent findings underscore the insufficient sensitivity of past screening methods [18]. As the condition was long regarded as affecting only motor function, the significance of cognitive-behavioural disorders was likely underestimated [2,26].

While previous broad analyses and meta-analyses have focused primarily on demographic and classical motor parameters like sex, site, age of onset, and survival, cognitive impairment could not be included quantitatively in those syntheses due to inconsistent reporting across published cohorts. Consequently, the principal contribution and novelty of the present review do not lie in merely demonstrating that cognitive and behavioural impairment can occur in *SOD1*-ALS, which is already established in isolated reports. Instead, this review specifically addresses the cognitive and behavioural spectrum in relation to individual *SOD1* variants, aiming to determine whether the apparent overall cognitive sparing traditionally attributed to *SOD1*-ALS conceals heterogeneous or variant-specific phenotypes.

This review aims to critically analyze and stratify data from the literature, integrating biological, neurophysiological, and clinical evidence associated with various *SOD1* gene mutations. The fundamental objective is to move beyond the traditional view of ALS as a disease confined exclusively to the motor system. Instead, the aim is to foster a detailed characterization of the neuropsychological profile of patients with *SOD1* mutations—rigorously integrated with their specific genetic background—thereby laying the groundwork for a truly personalized clinical and care approach.

The advent of gene-targeted therapies has substantially prolonged survival and altered the disease course of patients carrying *SOD1* mutations, thereby creating new opportunities to investigate the extent and evolution of cognitive involvement in this population. As disease-modifying treatments continue to extend life expectancy, longitudinal studies will be essential to determine whether cognitive dysfunction emerges or progresses over time, and to characterize its trajectory within the natural history of treated *SOD1*-related ALS.

## 2. Materials and Methods

### 2.1. Search Strategy

A systematic bibliographic search was conducted in accordance with the Preferred Reporting Items for Systematic Reviews and Meta-Analyses (PRISMA) guidelines. The search was performed in the PubMed database by two researchers (FG, FN) on 5 May 2026, using the following terms and search combinations: (“Amyotrophic Lateral Sclerosis”[mh] OR “Amyotrophic Lateral Sclerosis” OR ALS OR “Motor Neuron Disease”[mh] OR “MND”) AND (“ALS-FTD” OR “Frontotemporal Dementia”[mh] OR “Frontotemporal Dementia” OR FTD OR “Dementia”[mh] OR dement* OR cognitiv* OR behavior* OR neuropsycholog* OR “executive function*” OR “executive dysfunction” OR behavio* OR neuropsychiatr* OR apathy OR “Depression”[mh] OR depression OR “Anxiety”[mh] OR anxiety OR social cognition OR social behavior* OR “Language”[mh] OR Language OR “verbal fluency” OR memor* OR psychiatric OR “Agnosia”[mh] OR “visuospatial”) AND (“*SOD1*” OR “Superoxide Dismutase-1”[mh] OR “Superoxide Dismutase 1” OR “*SOD-1*”) NOT (animals[mh] NOT humans[mh]).

No chronological or methodological filters were applied to the database, apart from filtering by titles, keywords, and abstracts.

### 2.2. Inclusion and Exclusion Criteria

We included articles that met the following criteria: (1) patients with a clinical diagnosis of Amyotrophic Lateral Sclerosis (ALS) or Motor Neuron Disease (MND) variants, and/or frontotemporal dementia, (2) documented presence of a pathogenic variant in the *SOD1* gene, (3) studies reporting data on cognitive and behavioural status, neuropsychological testing, or the presence of frontotemporal dementia (FTD) symptoms. At the patient level, all *SOD1* carriers reported in an eligible study were extracted, irrespective of whether they exhibited cognitive or behavioural abnormalities; carriers with normal cognition and behaviour were retained in the dataset and are reported as such in the Results, rather than being excluded from extraction.

Exclusion criteria included articles without abstract or full text, reviews, articles about non-human models (in vitro studies, yeast models, or animal models (e.g., *SOD1*-p.Gly93Ala transgenic mice)), genetic reports that identify mutations but do not provide corresponding phenotypic or clinical data, studies in which cognitive or behavioural screenings were not performed, or that failed to report specific data on cognitive and behavioural phenotypes, and multiple reports of the same patient cohort (keeping only the most recent or complete study).

### 2.3. Study Selection

Following the initial search phase, the records obtained were imported in a CSV format into the Rayyan web application for blind screening. The researchers screened the articles in blind mode, based on title and abstract, followed by a full-text review, according to the aforementioned inclusion and exclusion criteria. At the end of the selection process, any discrepancies or conflicts between the two researchers regarding study eligibility were resolved through discussion. If consensus could not be reached, a third independent reviewer (LB) was consulted to make the final decision.

A total of 836 records were identified through the database search in PubMed. Following the removal of 5 duplicate records, 831 records underwent title and abstract screening. At this stage, 793 articles were excluded (782 based on title and 11 based on abstract). Of the 38 reports sought for retrieval, 1 could not be retrieved. The remaining 37 full-text reports were assessed for eligibility, and 20 were subsequently excluded.

**Figure 1:**
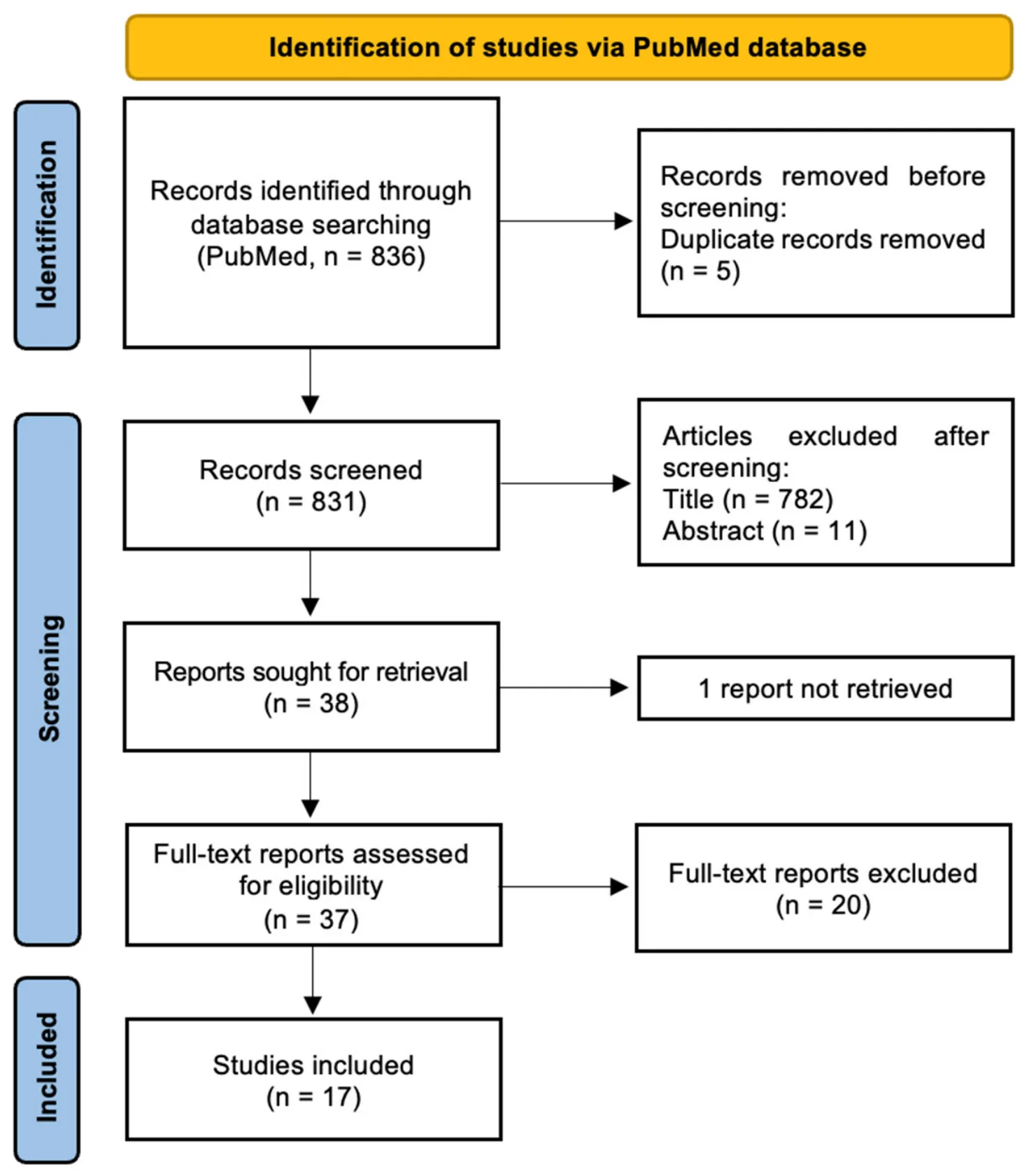
PRISMA (Preferred Reporting Items for Systematic Reviews and Meta-Analyses) flow diagram.

Ultimately, 17 studies met the eligibility criteria and were included in the present review (Figure 1).

### 2.4. Data Extraction

Data extraction was performed systematically by the two researchers using an item grid. The following information was extracted from each article; where available, individual case-level data were extracted; if strictly aggregated, data were grouped by mutation type: bibliographic details (first author’s name, article title, publication year), demographic and clinical information (number of patients, sex, family history of neurodegenerative diseases, onset symptom (motor, cognitive, behavioural, or mixed), age at motor and/or cognitive/behavioural symptom onset, specific motor features, clinical diagnosis (e.g., ALS, ALS-FTD, FTD, ALSci, ALSbi, ALScbi), genetic profile (*SOD1* variants, number of cases per variant, zygosity and inheritance pattern, screening for other genes), neuropsychological and instrumental assessment (specific cognitive domains impaired or behavioural symptoms, neuropsychological tests administered (e.g., MMSE, ECAS), applied diagnostic criteria for ALS-FTD). *SOD1* variants were reported according to the Human Genome Variation Society (HGVS) nomenclature, with numbering based on the reference sequences NM_000454.5 (mRNA) and NP_000445.1 (protein); historical (non-HGVS) variant names used in the original publications are provided in parentheses at first mention.

### 2.5. Quality Assessment and Risk of Bias

The methodological quality and risk of bias of the included studies were assessed independently by two reviewers. Given the heterogeneity of the included literature, critical appraisal was conducted using the appropriate Joanna Briggs Institute (JBI) critical appraisal checklists based on the specific study design (specifically, the checklists for case reports, case series, prevalence studies and cross-sectional studies). Disagreements were resolved by consensus or by involving a third reviewer. All included studies demonstrated a low risk of bias (see Supplementary Table S1). Checklist items scored “yes” were tallied for each study to summarize reporting quality at the item level: studies with ≥75% of applicable items scored “yes” were descriptively labelled as low risk, those with 50–74% as moderate risk, and those below 50% as high risk. This percentage-based labelling is intended as a descriptive summary of item-level responses rather than a validated risk-of-bias classification, as JBI checklists do not themselves specify a percentage cut-off for converting checklist scores into risk categories.

### 2.6. Data Analysis

Due to the heterogeneity across the included studies, as well as the lack of specific demographic, epidemiological and clinical data (often available only in aggregated form or small case reports), a formal meta-analysis could not be performed. Therefore, the data analysis was mainly descriptive.

Continuous variables (such as age at onset or neuropsychological test scores) and categorical variables were extracted and reported exactly as presented in the original studies. Any statistical comparisons, effect sizes, or *p*-values mentioned in the Results section reflect the primary statistical analyses conducted by the authors of the respective original papers.

## 3. Results

### 3.1. General Characteristics of Selected Studies

The studies analyzed in this review were published between 2001 and 2024. They included cohort studies, retrospective studies, cross-sectional studies, case series and case reports. Seventeen studies investigating the impact of *SOD1* mutations on cognitive symptoms in patients with ALS were selected. Participants enrolled had a confirmed diagnosis of ALS and at least one patient in these studies fulfilled one of the following criteria: a diagnosis of frontotemporal dementia (FTD); symptoms belonging to the ALS-frontotemporal spectrum with behavioural impairment (ALSbi), ALS with cognitive impairment (ALSci), or ALS with combined cognitive and behavioural impairment (ALScbi); or evidence of statistically significant cognitive deficits on standardized neuropsychological assessments compared with either healthy controls or patients with ALS who did not carry *SOD1* mutations. Across these studies, a total of 222 patients carrying *SOD1* mutations were investigated: 126 subjects exhibited no cognitive or behavioural abnormalities, thirteen were classified as having ALSbi, seven as ALSci, and two as ALScbi. Three fulfilled the criteria for ALS-FTD (this representing a descriptive count), specifically one patient in Müller et al. [27] met the criteria for early bvFTD, one in Nakamura et al. [28] fulfilled criteria for ALS-FTD, and one in Katz et al. [29] presented clinical features consistent with the behavioural or frontal variant of FTD. Among the remaining 71 individuals, nine presented with individually documented cognitive or behavioural abnormalities: one patient in Lopate et al. [30] demonstrated cognitive decline; one in Masè et al. [31] presented rapidly progressive cognitive deterioration with frontal lobe dysfunction; two in Martinelli et al. [32] showed impairment across multiple neuropsychological subtests; one in Battistini et al. [33] developed a syndrome characterized by visual hallucinations and mental confusion, whereas a second exhibited a frontal lobe-type disturbance; one in Canosa et al. [34] demonstrated significant frontopolar hypometabolism; one in Synofzik et al. [35] showed mild cognitive impairment; and one in Gagliardi et al. [36] displayed extrapyramidal signs alongside multidomain cognitive deficits, including ideomotor apraxia and reduced verbal fluency and comprehension. The remaining participants contributed exclusively to group-level statistical comparisons rather than presenting individual clinical diagnoses. Specifically, 25 subjects in Winroth et al. [25] showed statistically significant differences relative to healthy controls in tasks involving working memory, attention, and executive functions; 27 in Marjanović et al. [23] scored lower on the MMSE and the executive Stroop test compared to controls; seven in Wicks et al. [22] exhibited higher overall emotional lability as measured by the ELQ total score; and three in Niu et al. [37] demonstrated moderate impairment across several cognitive domains compared with healthy family members.

The mean age of the participants was approximately 54 years. Among the aforementioned studies, several had cohort sizes ranging from 24 to 956 participants, whereas others consisted of single case reports or small case series with sample sizes ranging from two to seven participants.

The most frequently investigated mutation was p.Asp91Ala, evaluated across six studies involving thirty-three patients. The p.Ile114Thr mutation was the focus of three studies involving eighteen patients. Additionally, a variety of less common mutations were evaluated across different studies: p.Ala89Val (A89V), p.Gly93Ser (G93S), p.Asp101Gly (D101G), p.Ser105Leu (S105L), p.Asp109Tyr (D109Y), p.Ile113Phe (I113F), p.Gly114Ala (G114A), p.Leu39Val (L39V), p.His44Arg (H44R), p.His47Arg (H47R), p.His49Arg (H49R), p.Gly73Ser (G73S), p.Leu85Phe (L85F), p.Asn87Ser (N87S), p.Val88Ala (V88A), p.Glu101Lys (E101K), p.Ile105Phe (I105F), p.Gly109Val (G109V), p.Ile114Thr (I114T) p.Arg116Gly (R116G), p.Glu134Lys (E134K), p.Leu145Phe (L145F), p.Gly148Asp (G148D), p.Val149Ala (V149A), p.Val149Gly (V149G), p.Ile150Thr (I150T), p.Gly141Ter (G141X), p.Leu144Phe (L144F), p.Ala145Gly (A145G), p.Asn66Thr (N66T), p.Ser26Asn (S26N), p.Asn66Ser (N66S), p.Gly94Asp (G94D), p.Gly12Arg (G12R), p.Gly41Ser (G41S), p.Ser59Ser (S59S), p.Gly147Cys (G147C), p.Gly37Arg (G37R), p.Leu8Val (L8V), p.Asp76Tyr (D76Y), p.Glu100Gly (E100G), p.Gly93Ala (G93A), p.Gly41Asp (G41D), p.Gly13Arg (G13R), p.Glu22Gly (E22G), p.Gln23Arg (Q23R), p.Pro67Leu (P67L), p.Pro67Ser (P67S), p.Ala96Thr (A96T), p.Leu107Val (L107V), p.Leu118Val (L118V), p.Glu122Gly (E122G), p.Leu145Ser (L145S), p.Ala5Val (A5V), p.Asn20Ser (N20S), p.Gly42Ser (G42S), p.Phe46Cys (F46C), p.Gly62Arg (G62R), and p.Asp110Tyr (D110Y).

A detailed description of all study and patient characteristics is provided in Table 1.

#### 3.1.1. Evaluation of Global Cognition

Overall, the available results suggested that carriers of *SOD1* mutations may exhibit global cognitive impairment, although the severity and clinical presentation appear to vary across mutation types. Specifically, some studies reported lower Mini-Mental State Examination (MMSE) scores in *SOD1* patients compared to healthy controls. Moreover, one study highlighted a progressive decline in MMSE scores over time, associated with rapid and progressive cognitive deterioration and frontal lobe dysfunction, particularly in carriers of the p.Leu144Phe variant. Mild cognitive impairment has been observed in patients with the p.Gly93Ala mutation, as documented by an MMSE score of 25/30 and Detect scores of 9/18. Carriers of other variants, including p.Gly41Asp, presented a more severe cognitive impairment affecting multiple cognitive domains, corresponding to an MMSE score of 20/30. Finally, across the included studies, 25 patients were diagnosed with a defined cognitive or behavioural syndrome within the ALS-frontotemporal spectrum, including thirteen patients with ALSbi, seven with ALSci, and two with ALScbi. In addition, three patients fulfilled the diagnostic criteria for ALS-FTD.

#### 3.1.2. Evaluation of Language

Within the language domain, significant deficits have been detected in several patients with various *SOD1* mutations. Some of the homozygous carriers of the p.Asp91Ala variant displayed alterations in Listening Span and in the Controlled Oral Word Association Test (COWAT). A mild reduction in verbal fluency and constrained lexical abilities was reported in carriers of the p.His49Arg and p.Asn66Thr variants. Patients with the p.Ile114Thr mutation showed more severe language impairment, characterized by reduced spontaneous speech and word-finding difficulties, which in some cases progressed to hypokinetic mutism. Specific assessments confirmed severe language impairment, evaluated for instance via the Chinese version of the Boston Naming Test (C-BNT), coupled with a deterioration in both phonemic and semantic verbal fluency. Additional case series documented a marked reduction in verbal fluency and comprehension, despite relatively preserved short-term memory.

#### 3.1.3. Evaluation of Visuospatial Skills

The neuropsychological tests commonly used to assess visuospatial abilities were the Rey–Osterrieth Complex Figure Test (ROCF, or RCFT) and the Clock Drawing Test. Some patients carrying *SOD1* mutation variants, including p.Asp91Ala and p.Gly147Cys, demonstrated significant impairments in both the copy and recall of the Rey–Osterrieth Complex Figure Test compared to controls. Similarly, performance on the Clock Drawing Test was consistently poorer in *SOD1*-associated ALS patients compared to healthy control groups.

#### 3.1.4. Evaluation of Executive Functions

Among the several patients evaluated, executive dysfunction was one of the most consistently reported cognitive features in patients with *SOD1* mutations. Deficits were identified with specific neuropsychological batteries including the Stroop test and the Frontal Assessment Battery (FAB). Executive impairment was also reflected by reduced scores on the executive domain of the Edinburgh Cognitive and Behavioural ALS Screen (ECAS), indicating deficits in mental control and higher-order executive processes. Additional impairments were observed on the trail making test (TMT-A and TMT-B), suggesting reduced cognitive flexibility, processing speed, and set-shifting abilities.

From a clinical perspective, executive dysfunction manifested with ideomotor apraxia, mental rigidity, and perseverative behaviour. Furthermore, performance on tests of non-verbal reasoning and general intellectual functioning, such as the Raven’s Coloured Progressive Matrices (CPM-47), was significantly poorer in patients with *SOD1*-associated ALS than in healthy controls.

#### 3.1.5. Evaluation of Memory

Working memory and visual memory impairments were frequently reported in *SOD1* mutation carriers. Deficits in working memory were quantitatively identified using specific tests including the Digit Span Forward (FW) and Backward (BW) tests. Neuropsychological evaluations revealed reduced performance in delayed recall tests, evidenced by poor scores on the ROCF delayed recall and on the Hopkins Verbal Learning Test (HVLT). Despite deficits across multiple cognitive domains, several studies reported relatively preserved short-term memory in some patients, suggesting that immediate memory span may remain intact even in the presence of more widespread cognitive impairment.

#### 3.1.6. Evaluation of Attention and Processing Speed

Patients carrying various *SOD1* gene variants also showed impairment in attention and concentration capacities compared to healthy controls. In specific case series (e.g., in patients with the p.Gly41Asp variant), although the attention domain was globally considered compromised, sub-scores on the Digit Span Forward sometimes appeared similar between the ALS group and healthy family members.

#### 3.1.7. Evaluation of Psychiatric Symptoms

Psychiatric manifestations were reported less frequently than cognitive and behavioural disturbances. Nevertheless, they were documented in carriers of specific *SOD1* variants. Isolated case reports described the occurrence of perceptual disturbances, including visual hallucinations, as well as episodes of severe confusional states during disease progression.

#### 3.1.8. Behaviour and Emotion

Behavioural disturbances and emotional dysregulation were among the most prominent non-motor manifestations reported in a subset of patients carrying *SOD1* mutations. According to the Strong criteria, several patients fulfilled the diagnostic criteria for ALS with behavioural impairment (ALSbi), presenting symptoms such as irritability, mental rigidity, and behavioural disinhibition, including inappropriate sexual behaviour. Compared with healthy controls, some cohorts also exhibited higher levels of apathy and emotional blunting.

Emotional dysregulation was further characterized by pathological crying (emotional lability) and episodes of affective instability, reflected by higher total scores on the Emotional Lability Questionnaire (ELQ). In the most severe cases, the behavioural profile resembled that of the behavioural variant of frontotemporal dementia (bvFTD), with manifestations including aggression, profound social disinhibition, and markedly inappropriate or irrational behaviours. For example, one patient was reported to wander unclothed at night while chasing household pets. Severe behavioural impairment was further supported by markedly reduced performance on the ALS cognitive behavioural screen (ALS-CBS), with one patient obtaining a behavioural score of 0/20.

**Figure 2:**
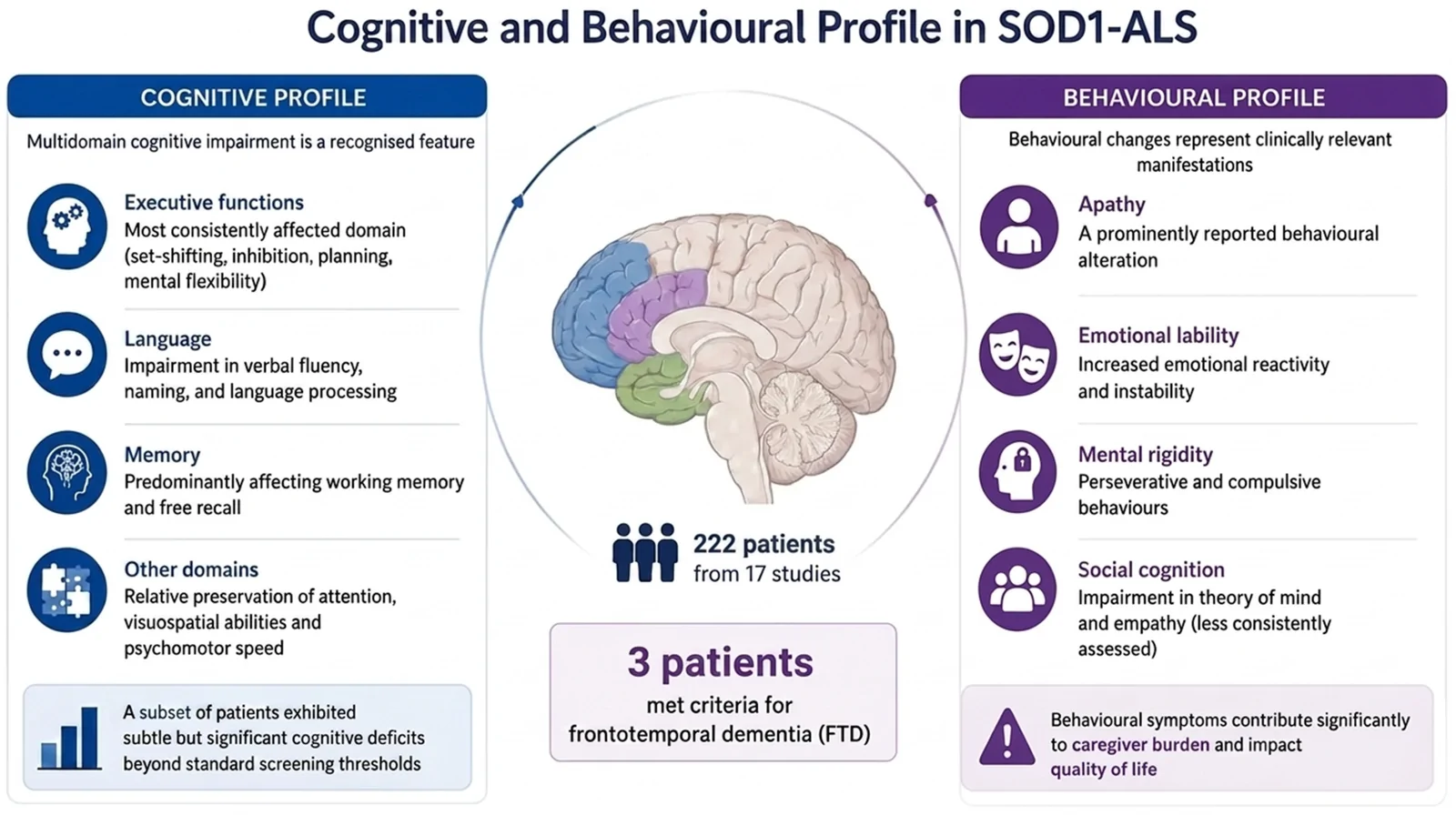
Summary of the cognitive and behavioural profile in *SOD1*-ALS. This figure provides an overview of the main cognitive and behavioural domains affected in patients with *SOD1*-related ALS, as identified in the 17 studies included in this systematic review (222 patients).

Overall, the available evidence supports the concept that *SOD1*-ALS encompasses a heterogeneous spectrum of cognitive and behavioural manifestations. Figure 2 summarizes the principal affected domains and the main behavioural features identified across the studies included in our systematic review of the literature.

## 4. Discussion

Historically, ALS cases associated with *SOD1* mutations were regarded as predominantly restricted to motor manifestations, with limited or absent involvement of cognitive and behavioural domains [38].

In contrast, mutations in other ALS-associated genes, including *C9orf72*, *TARDBP* and *TBK1*, have been frequently linked to cognitive and behavioural impairment in patients, with clinical manifestations ranging from subtle deficits to overt frontotemporal dementia (FTD) [19].

The findings of this systematic review challenge the traditional view of *SOD1*-related ALS as a predominantly motor disorder. Our results provide evidence that, although less prevalent than in other genetically determined forms of ALS, cognitive and behavioural impairments represent relevant components of the clinical spectrum associated with *SOD1* mutations, extending the phenotype into the ALS-frontotemporal dementia (ALS-FTD) continuum.

Specifically, three of the analyzed subjects (a descriptive count, not an estimate of prevalence) fulfilled the diagnostic criteria for FTD [2] or its behavioural variant (bvFTD) [39]. Moreover, an even larger proportion of patients exhibited multidomain cognitive impairment. The main *SOD1* pathogenic variants identified in patients presenting with phenotypic features consistent with FTD included p.Gly141Ter [28], p.His49Arg [27] and p.Ile114Thr [29].

In this context, our review provides a distinct contribution compared to previous broad analyses of the *SOD1*-ALS phenotype. While prior comprehensive studies were restricted to classical clinical variables, our work specifically dissects the non-motor spectrum across individual *SOD1* variants. The main strength of this approach is not simply confirming the occurrence of cognitive impairment in *SOD1*-ALS, but rather demonstrating that the apparent overall cognitive sparing observed at a group level actually conceals a highly heterogeneous scenario characterized by variant-specific cognitive and behavioural phenotypes.

Nakamura et al. [28] reported a case of ALS-FTD associated with the p.Gly141Ter *SOD1* variant in a patient with a strong family history of ALS.

The p.His49Arg variant was identified in a comprehensive analysis of 301 German families [27], including a patient who presented without motor involvement and whose initial clinical manifestations were characterized by aggression, emotional lability, reduced working memory and impaired verbal fluency, features consistent with a behavioural variant of frontotemporal dementia (bvFTD). While this might appear as an atypical presentation of *SOD1*-associated disease, the presence of a cerebrospinal fluid biomarker profile suggestive of Alzheimer’s disease (AD) pathology introduces a critical interpretative challenge. On one hand, the AD biomarker profile may reflect a concomitant, independent neurodegenerative process acting as a major confounding factor; in this view, the cognitive decline cannot be directly attributed to the *SOD1* variant. On the other hand, it raises the intriguing hypothesis that specific *SOD1* mutations might directly trigger, intersect with, or exacerbate an AD-like pathological cascade. Ultimately, this case highlights the complexity of differentiating two pathologies from true phenotypic expansions when evaluating atypical cognitive phenotypes in ALS cohorts.

The p.Ile114Thr variant was described in a case report [29] involving a patient who presented with progressive language impairment characterized by reduced spontaneous speech and word-finding difficulties, followed by behavioural changes including disinhibition and apathy. Notably, these cognitive and behavioural manifestations preceded the onset of motor symptoms by approximately two years, suggesting that even in a subset of *SOD1*-related cases, non-motor symptoms may emerge before the development of classical motor manifestations.

Overall, these results highlight that cognitive and behavioural impairment is relevant in *SOD1* mutations, even in the earlier stages of the disease.

When considering the cognitive and behavioural domains affected in our cohort and comparing these findings with recent systematic reviews, a complex and heterogeneous neuropsychological profile emerges. A recent meta-analysis by Jiménez-García et al. [40] suggested that patients with *SOD1* mutations generally exhibit a relatively preserved cognitive profile compared with carriers of other ALS-associated genes, with no significant impairments identified in memory, language, or executive functions.

In contrast, our findings indicate a broader and more heterogeneous spectrum of cognitive and behavioural involvement, demonstrating that specific cognitive domains and behavioural features may be significantly impaired even in the absence of a formal diagnosis of frontotemporal dementia (FTD).

These results are consistent with the review by Martinelli et al. [19], which highlighted emerging evidence of frontal lobe network dysfunction in *SOD1* mutation carriers, challenging the historical assumption that these neural networks are entirely preserved in this genetic form of ALS.

Our systematic review highlighted the presence of executive dysfunction in a subset of *SOD1* mutation carriers, characterized by impairments in mental control, cognitive flexibility, and problem-solving abilities. In a large cohort study, Calvo et al. [18] reported significantly poorer performances on the Frontal Assessment Battery (FAB) and the Clock Drawing Test compared with healthy controls, supporting the involvement of frontal-executive networks in *SOD1*-associated ALS. Similarly, Marjanović et al. [23] identified marked deficits on the executive component of the Stroop test, indicating impaired inhibitory control and reduced cognitive flexibility.

Clinically, this dysexecutive profile may manifest as mental rigidity and perseverative behaviour, as described by Katz et al. [29] and Dalla Bella et al. [24]. Cognitive impairment in these patients may extend beyond isolated frontal-executive dysfunction, involving multiple cognitive domains and, in severe cases, being associated with manifestations such as ideomotor apraxia, as reported in both familial cohorts and isolated case reports [29,36].

Deficits in sustained attention and concentration have also been documented. Niu et al. [37] described a heterogeneous pattern of executive impairment, in which global attentional performance and backward digit span were significantly reduced, whereas other components, such as forward digit span, remained relatively preserved and comparable to those observed in healthy family members.

Similarly, while language was previously considered to be generally preserved in the *SOD1* population [40], our review documents a broad spectrum of linguistic deficits. Impairments range from a mild reduction in verbal fluency and constrained lexical abilities to more severe linguistic deterioration. Niu et al. [37] observed significant language impairments using the Chinese version of the Boston Naming Test (C-BNT), and Gagliardi et al. [36] documented a marked reduction in verbal fluency and comprehension. Calvo et al. [18] registered poor performance in the ECAS Verbal Fluency subdomain for the *SOD1*-ALS patients compared to patients with sALS. In a dramatic case reported by Katz et al. [29], the patient experienced a gradual reduction in spontaneous speech and prominent word-finding difficulties that ultimately progressed to complete mutism within three years, notably despite relatively preserved bulbar function.

Our results also challenge the concept that memory and visuospatial networks are universally preserved. Comparing the *SOD1*-ALS patients to healthy controls, deficits in working memory and visual memory have been quantitatively identified by Calvo et al. [18] using tools such as the Digit Span Backward and the Rey–Osterrieth Complex Figure (ROCF) delayed recall. However, in line with the marked heterogeneity of the disease, short-term memory may remain paradoxically intact in some patients despite a severe multidomain decline, as observed by Gagliardi et al. [36]. Furthermore, both Calvo et al. [18] and Canosa et al. [34] observed substantial deficits in tasks requiring visuospatial integration.

Regarding the behavioural and emotional profile, our results corroborate the existing literature. Emotional lability, apathy, irritability, and mental rigidity emerged as central pathological elements in a subset of *SOD1* phenotypes. These are consistent with those reported by Jiménez-García et al. [40], who highlighted that *SOD1* mutation carriers exhibit a distinctive emotional profile characterized by increased emotional lability and significant apathy. Moreover, Martinelli et al. [32] reported cases of *SOD1* patients presenting with aggression, emotional dysregulation and social behaviour disturbances, including sexual disinhibition and reduced social appropriateness.

Although less frequently reported, psychiatric manifestations also represent a relevant component of the clinical spectrum, with isolated cases documenting perceptual disturbances, visual hallucinations, and severe confusion states.

Overall, our review supports the concept that cognitive and behavioural involvement in *SOD1*-associated ALS extends beyond the presence of clinically defined frontotemporal dementia (FTD) [2]. Even in patients who retain relatively preserved global cognition or do not fulfil formal diagnostic criteria for FTD, specific behavioural and psychiatric symptoms may substantially affect daily functioning and quality of life.

From a genetic perspective, our data support the possible existence of genotype–phenotype correlations concerning non-motor symptoms in *SOD1* carriers. Among the 222 cases analyzed, the p.Asp91Ala and p.Ile114Thr mutations were the variants most associated with cognitive and behavioural impairment.

The homozygous p.Asp91Ala mutation, highly prevalent in Scandinavian populations [41], is usually associated with a very slow disease progression [25,42]. This prolonged disease course may facilitate the emergence of subtle cognitive changes, including mild executive, attentional and working memory impairments, which may remain undetected in ALS variants characterized by a more rapid clinical course [43].

This phenotypic profile is consistent with the hypothesis proposed by Martinelli et al. [19], suggesting that cognitive and behavioural impairments may represent a late manifestation of a long-standing neurodegenerative process. According to this framework, neurodegeneration may initially predominantly affect motor regions and progressively spread to the prefrontal areas, with cognitive and behavioural symptoms becoming clinically evident only after sufficient disease duration. Therefore, these manifestations may be more readily observed in slowly progressive phenotypes, whereas they may remain clinically silent in rapidly progressive forms with limited survival time [43].

Notably, mutations located in Exon 5 (spanning from codon 119 to 153), including the p.Gly141Ter described by Nakamura et al. [28], p.Leu144Phe described by Masè et al. [31], and p.Gly148Asp, p.Ile150Thr, and p.Leu145Phe described by Dalla Bella et al. [24], appeared to be strongly associated with early behavioural impairment and rapid frontal lobe dysfunction.

Similarly, the p.Ile114Thr variant was associated, in our extracted cases, with severe language impairment and mutism [29].

These findings may support the hypothesis by Martinelli et al. [19] that specific *SOD1* mutations may influence the anatomical distribution and progression patterns of neurodegeneration, contributing to the high clinical heterogeneity associated with *SOD1*-ALS.

The marked clinical heterogeneity observed across the 222 patients suggests that *SOD1*-associated ALS should not be considered a single homogeneous entity. The traditional assumption that *SOD1* mutations predominantly spare cognitive networks may, at least in part, reflect under-recognition of subtle cognitive and behavioural manifestations, resulting from both limited knowledge of non-motor symptoms and the reduced sensitivity of earlier screening approaches [44,45].

### 4.1. Limitations

Our findings should be interpreted in the context of several limitations, which derive from both the nature of the available literature and the specific methodological scope of this review.

#### 4.1.1. Limitations of the Included Evidence

Publication and reporting bias: the literature detailing cognitive and behavioural impairment in *SOD1* mutation carriers consists predominantly of case reports and small case series. This introduces a risk of publication bias, as clinicians are more likely to report unusual, severe, or unexpected phenotypes (e.g., overt FTD). Consequently, the frequency of these cognitive and behavioural phenotypes might be overrepresented in the current literature. However, this potential overrepresentation supports our choice to employ a purely descriptive approach to data synthesis rather than a meta-analysis. Furthermore, while the included studies demonstrated high reporting quality according to the JBI criteria (resulting in a low risk of bias classification), the inherent nature of individual case reports and small case series implies that the overall strength of evidence remains limited. This intrinsic design limitation restricts broad generalizability and highlights the need for larger, longitudinal cohort studies.

Methodological heterogeneity: we observed considerable heterogeneity across the included studies regarding the neuropsychological tools and diagnostic frameworks applied. Older reports frequently relied on generic screening instruments, such as the Mini-Mental State Examination (MMSE), which lack optimal sensitivity for detecting the specific executive and language deficits characteristic of the ALS-FTD spectrum. Furthermore, the evolution of diagnostic criteria over time (e.g., the transition from the 2009 to the 2017 Strong criteria) [2,7] implies that similar clinical profiles may have been classified differently depending on the publication year, complicating direct comparisons between older and more recent cohorts.

#### 4.1.2. Limitations of the Review Processes

Database selection: methodologically, our literature search was restricted to the PubMed database. Although this provided a comprehensive baseline, the omission of other major repositories such as Embase or Scopus leaves the possibility that some relevant records may have been inadvertently excluded.

Targeted inclusion criteria: in line with our primary objective of characterizing the variable expressivity of cognitive and behavioural phenotypes, we deliberately selected *SOD1* variants associated with these specific impairments, excluding those with exclusively motor presentations. While this targeted approach was essential for conducting an in-depth analysis of phenotypic variability, it prevents our dataset from being used to estimate the absolute epidemiological prevalence or incidence of cognitive and behavioural impairment across the *SOD1* mutation spectrum.

## 5. Conclusions

Our findings indicate that *SOD1* mutations should not be excluded from the genetic landscape associated with cognitive and behavioural impairment in ALS. Cognitive and behavioural manifestations have been documented in a heterogeneous subset of *SOD1*-ALS patients, and possible variant-specific associations require confirmation in appropriately designed cohorts. Recognizing and characterizing these manifestations is becoming increasingly relevant, particularly in the era of targeted disease-modifying therapies such as Tofersen. In this context, comprehensive neurocognitive assessment may represent a valuable tool for monitoring disease progression, identifying phenotypic trajectories, and evaluating therapeutic response.

## Figures and Tables

**Table 1:** Description of the different cognitive and behavioural changes in *SOD1*-ALS patients. Legend: for each study, the number of *SOD1* carriers with cognitive/behavioural impairment is further qualified as “formal diagnosis” (impairment meeting explicit diagnostic criteria for ALSbi/ALSci/ALScbi), “individually documented” (case-level abnormalities described for named individuals without formal criteria applied), or “group-comparison only” (the entire subgroup counted on the basis of a statistically significant group-level difference versus controls or non-carriers, without individual-level clinical diagnosis).

Study Label	*SOD1* Cases	*SOD1* Carriers with Cognitive/Behavioural Impairment	*SOD1* Variants Identified	Cognitive and Behavioural Changes
Winroth et al. (2024) [25]	25	25 (group-comparison only)	p.Asp91Ala, p.Ala5Val, p.Ala89Val, p.Gly93Ser, p.Asp101Gly, p.Ser105Leu, p.Asp109Tyr, p.Ile113Phe, p.Gly114Ala.	Differences between healthy controls and ALS patients in tasks involving working memory, attention, and executive functions. Homozygous carriers of p.Asp91Ala *SOD1* showed deficits regarding Listening Span, Mental Control, COWAT, and RCFT Copy. [Diagnostic criteria/instruments: Listening Span, Mental Control, COWAT, RCFT Copy.]
Montuschi et al. (2014) [5]	5	1 (individually documented)	Unspecified	1 case of ALSbi. [Diagnostic criteria/instruments: Strong criteria (ALSbi); specific instrument not stated in source.]
Müller et al. (2018) [27]	37	1 (individually documented)	p.Leu39Val, p.His44Arg, p.His47Arg, p.His49Arg, p.Gly73Ser, p.Leu85Phe, p.Asn87Ser, p.Val88Ala, p.Asp91Ala, p.Glu101Lys, p.Ile105Phe, p.Gly109Val, p.Ile114Thr, p.Arg116Gly, p.Glu134Lys, p.Leu145Phe, p.Gly148Asp, p.Val149Ala, p.Val149Gly, p.Ile150Thr.	One patient with the p.His49Arg mutation presented symptoms that were consistent with an early bvFTD (aggression, emotional lability, reduced working memory and slightly reduced verbal fluency). At the same time, CSF analysis was in agreement with Alzheimer’s disease (increased tau and decreased amyloid-β values). [Diagnostic criteria/instruments: Clinical bvFTD criteria; specific instrument not stated in source.]
Nakamura et al. (2015) [28]	7	1 (individually documented)	p.Gly141Ter	1 case ALS-FTD. [Diagnostic criteria/instruments: ALS-FTD criteria; specific instrument not stated in source.]
Lopate et al. (2010) [30]	15	1 (individually documented)	p.Ile114Thr	One case of cognitive decline. [Diagnostic criteria/instruments: Criteria/instrument not specified in source.]
Marjanović et al. (2017) [23]	27	27 (group-comparison only)	p.Asp91Ala, p.Leu144Phe, p.Ala145Gly	MMSE scores were lower in the *SOD1* group compared to healthy controls (HCs) (*p* < 0.01). *SOD1* positive patients achieved a significantly lower score on the executive Stroop test than HCs (*p* < 0.01). [Diagnostic criteria/instruments: MMSE; executive Stroop test.]
Masè et al. (2001) [31]	18	1 (individually documented)	p.Leu144Phe	One patient presented a rapidly progressive cognitive deterioration with characteristics of frontal lobe dysfunction. [Diagnostic criteria/instruments: Criteria/instrument not specified in source.]
Martinelli et al. (2020) [32]	2	2 (individually documented)	p.Asn66Thr	In one patient, the ECAS test (Italian version) showed an impairment in executive functions. ECAS total score was 89/136. The patient showed predominant behavioural disinhibition and emotional lability. In a second patient, an extensive neuropsychological assessment including exploration of memory, language, attention and executive functions revealed limitations in lexical abilities and mildly compromised executive functions. [Diagnostic criteria/instruments: Edinburgh Cognitive and Behavioural ALS Screen (ECAS).]
Dalla Bella et al. (2022) [24]	14	9 (formal diagnosis)	p.Asp91Ala, p.Gly148Asp, p.Ile150Thr, p.Ser26Asn, p.Asn66Ser, p.Gly73Ser, p.Gly94Asp, p.Leu145Phe	6 ALSbi, 2 ALSci, 1 ALScbi. Behavioural impairment defined by the Strong criteria, and most commonly featuring irritability and mental rigidity, was more frequent in *SOD1*+ than *SOD1*− patients and mainly associated with variants in Exon 5. [Diagnostic criteria/instruments: Strong criteria (ALSbi/ALSci/ALScbi).]
Battistini et al. (2014) [33]	7	2 (individually documented)	p.Gly12Arg, p.Leu144Phe, p.Gly41Ser, p.Asp91Ala, p.Ser59Ser	One patient had a visual hallucination syndrome and mental confusion. A second patient developed a behavioural derangement characterized by sexual disinhibition, after nine months of illness. The mental status examination showed a disturbance of the frontal lobe type. [Diagnostic criteria/instruments: Criteria/instrument not specified in source.]
Canosa et al. (2014) [34]	3	1 (individually documented)	p.Gly147Cys	The proband on the neuropsychological evaluation showed an impaired performance in the Rey–Osterrieth Complex Figure (ROCF) Test, in the copy and recall tasks. Neuropsychological assessments were repeated 6 months later, which confirmed the deficit in the ROCF and demonstrated a reduction in the scores of the Mini-Mental State Examination (MMSE), trail making test (TMT)-A and TMT-B, the Clock Test and Frontal Assessment Battery (FAB), although they were still normal. 18F-FDG PET showed the appearance of an area in the right frontopolar region (*p* = 0.01). A relative reduction in the uptake in the right caudate nucleus, probably due to deafferentation, seems to support the significance of the frontopolar hypometabolism. [Diagnostic criteria/instruments: MMSE, TMT-A/B, Clock Test, ROCF.]
Wicks et al. (2009) [22]	7	7 (group-comparison only)	p.Gly37Arg, p.Leu8Val, p.Asp76Tyr, p.Ile114Thr, p.Glu100Gly	*SOD1* FALS patients had higher levels of apathy (U = 61.5, *p* = 0.038). Relative to control participants, there was a trend approaching significance for higher levels of labile crying amongst *SOD1* FALS patients (U = 65.0, *p* = 0.053). In comparison to control participants, *SOD1* FALS patients (U = 40.5, *p* = 0.004) had higher levels of overall lability as measured by the ELQ Total score. [Diagnostic criteria/instruments: Emotional Lability Questionnaire (ELQ).]
Calvo et al. (2024) [18]	28	12 (formal diagnosis)	p.Ala5Val, p.Asn20Ser, p.Gly42Ser, p.Phe46Cys, p.Gly62Arg, p.Asn66Ser, p.Gly73Ser, p.Leu85Phe, p.Gly94Asp, p.Asp110Tyr, p.Leu145Phe, p.Ile150Thr	Among the 28 *SOD1* patients, 16 (57.1%) had normal cognitive function, five had ALSci (17.9%), six had ALSbi (21.4%), and one had ALScbi (3.6%). Compared to controls, *SOD1*-ALS patients showed poorer performance in tests assessing executive functions (FAB [*p* = 0.0001], ROCF-IR [*p* = 0.001], and Clock Drawing Test [*p* = 0.001]), attention and working memory (Digit Span FW and Digit Span BW [both *p* = 0.0001]), visual memory (ROCF-DR) and non-verbal general intelligence (CPM47 [*p* = 0.01]). SET-IA (*p* = 0.009), SET-EA (*p* = 0.003) and SET-GS (*p* = 0.01) scores were lower in *SOD1*-ALS patients compared to controls. In addition, the MMSE score was lower in *SOD1*-ALS patients (*p* = 0.041). [Diagnostic criteria/instruments: Strong criteria (ALSci/ALSbi/ALScbi); FAB, ROCF, Clock Drawing Test, Digit Span.]
Synofzik et al. (2010) [35]	4	1 (individually documented)	p.Gly93Ala	One patient showed mild cognitive impairment yielding twenty-five out of thirty points on the Mini-Mental State Examination and nine out of eighteen points on the Detect, a psychometric screening test that is sensitive for identifying patients with dementia, especially in the initial stages of the disease. [Diagnostic criteria/instruments: MMSE; Detect.]
Katz et al. (2012) [29]	1	1 (individually documented)	p.Ile114Thr	A 54-year-old male developed paucity of speech and word-finding difficulty, increased disinhibition and later increased apathy and emotional blunting. Subsequently he developed severe aphasia. 3 years after symptom onset, he was mute despite relatively preserved bulbar function and only nodded in response to simple questions. He had difficulty executing one-step commands and showed marked apraxia and perseveration of his actions. He could sign his name but no other words. His clinical features were considered consistent with the behavioural or frontal variant of FTD. His score on the ALS cognitive behavioural screen (ALS-CBS), used to screen for FTD in patients with ALS, was zero out of a possible 20 (normal, greater than 16). [Diagnostic criteria/instruments: Clinical bvFTD criteria; specific instrument not stated in source.]
Niu et al. (2016) [37]	3	3 (group-comparison only)	p.Gly41Asp	Neuropsychological testing revealed moderate impairment in some cognitive domains. The proband’s MMSE score was 20 (of 30). The executive domain, attention domain, language function, calculation tasks and memory were significantly impaired in the patients with ALS compared to the healthy family members. The executive domain, as assessed using the backward digit span, category verbal fluency, phonemic verbal fluency and Stroop tests, as well as the attention domain, were significantly impaired in the ALS group. The attention domain was examined by forward digit span, and the scores of the ALS and healthy groups were similar. In terms of language function measured using the Chinese version of the Boston Naming Test (C-BNT), the ALS group was significantly impaired compared to the healthy group. In calculation tasks and memory, the Chinese version of the Hopkins Verbal Learning Test (CVLT) delayed recall scores, and the Rey complex figure test (RCFT) delayed recall scores were significantly lower in the ALS group (Fisher’s exact test, *p* < 0.01). [Diagnostic criteria/instruments: MMSE, Digit Span, RCFT, executive Stroop test.]
Gagliardi et al. (2023) [36]	19	1 (individually documented)	p.Asp91Ala, p.Gly13Arg, p.Glu22Gly, p.Gln23Arg, p.Pro67Leu, p.Pro67Ser, p.Ala96Thr, p.Leu107Val, p.Leu118Val, p.Glu122Gly, p.Leu145Phe, p.Leu145Ser	One patient was hospitalized due to a history of unexplained and repeated falls, by the age of 55 years. Neurological examination showed occasional fasciculations in the calves (confirmed by EMG), with normal muscle strength and trophism and normal DTRs and flexor plantar responses. However, the patient was unable to walk without assistance due to a notable gait ataxia. The pull test was positive, and finger-to-nose and heel-to-knee tests were impaired. Mild bradykinesia and axial rigidity were present. His family members described behavioural changes occurring in recent years. Detailed neuropsychological evaluation evidenced severe, multidomain cognitive deficits, with ideomotor apraxia and reduced verbal fluency and comprehension despite relatively preserved short-term memory. [Diagnostic criteria/instruments: Criteria/instrument not specified in source.]

Abbreviations: ALS: Amyotrophic Lateral Sclerosis; ALSbi: ALS behavioural impairment; ALSci: ALS cognitive impairment; ALScbi: ALS cognitive and behavioural impairment; COWAT: Controlled Oral Word Association Test; RCFT Copy: Rey Complex Figure Test Copy; FTD: frontotemporal dementia; bvFTD: behavioural variant FTD; CSF: cerebrospinal fluid; MMSE: Mini-Mental State Examination; ECAS: Edinburgh Cognitive and Behavioural ALS Screen; FALS: familial ALS; ROCF-DR: Rey–Osterrieth complex figure–delayed recall; CPM47: coloured progressive matrices form 1947; DTR: deep tendon reflexes.

## Data Availability

No new data were created or analyzed in this study. Data sharing is not applicable to this article.

## Supplementary Materials

The following supporting information can be downloaded at: https://www.mdpi.com/article/10.3390/ijms27177958/s1.

## Abbreviations

The following abbreviations are used in this manuscript:

ALS	Amyotrophic Lateral Sclerosis
ALSbi	ALS behavioural impairment
ALSci	ALS cognitive impairment
ALScbi	ALS cognitive and behavioural impairment
COWAT	Controlled Oral Word Association Test
CPM47	Coloured Progressive Matrices form 1947
CSF	Cerebrospinal Fluid
DTR	Deep Tendon Reflexes
ECAS	Edinburgh Cognitive and behavioural ALS screen
FALS	Familial ALS
FTD	Frontotemporal Dementia
bvFTD	behavioural variant FTD
MMSE	Mini-Mental State Examination
RCFT Copy	Rey Complex Figure Test Copy
ROCF-DR	Rey–Osterrieth complex figure–delayed recall
