# Supplementary material for: Cognitive and Behavioural Profile of SOD1-ALS Across the ALS-FTD Spectrum: A Systematic Review
Source: Int J Mol Sci. 2026 Sep 7;27(17):7958. doi: 10.3390/ijms27177958 (PMC13565861; doi:10.3390/ijms27177958)
Supplement: Supplementary file 1 [file ijms-27-07958-s001.zip › ijms-4513317-supplementary.pdf]

Study Label	Study design	Q1	Q2	Q3	Q4	Q5	Q6	Q7	Q8	Q9	Q10	Overall risk of bias
Winroth et al. (2024)[25]	Cross-sectional study	Y	Y	Y	Y	Y	Y	Y	Y	-	-	Low
Montuschi et al. (2014) [5]	Cross-sectional study	Y	Y	Y	Y	Y	Y	Y	Y	-	-	Low
Müller et al. (2018) [27]	Cross-sectional prevalence study	Y	Y	Y	Y	Y	Y	Y	Y	-	-	Low
Nakamura et al. (2015) [28]	Case report	Y	Y	Y	Y	Y	NA	NA	NA	-	-	Low
Lopate et al. (2010) [29]	Case series	Y	Y	Y	Y	Y	Y	Y	Y	Y	N	Low
Marjanović et al. (2017) [23]	Cross-sectional study	Y	Y	Y	Y	Y	Y	Y	Y	-	-	Low
Masè et al. (2001) [30]	Case series	Y	Y	Y	Y	Y	Y	Y	Y	Y	Y	Low
Martinelli et al. (2020) [31]	Case series	Y	Y	Y	U	Y	Y	Y	Y	Y	NA	Low
Dalla Bella et al. (2022) [24]	Cross-sectional study	Y	Y	Y	Y	Y	Y	Y	Y	-	-	Low
Battistini et al. (2014) [32]	Prevalence study	Y	Y	Y	Y	Y	Y	Y	Y	NA	-	Low
Canosa et al. (2014) [33]	Case report	Y	Y	Y	Y	NA	NA	NA	Y	-	-	Low
Wicks et al. (2009) [22]	Cross-sectional study	Y	Y	Y	Y	Y	Y	Y	Y	-	-	Low

Calvo et al. (2024) [18]	Cross-sectional study	Y	Y	Y	Y	Y	Y	Y	Y	-	-	Low
Synofzik et al. (2010) [34]	Case series	Y	Y	Y	Y	Y	Y	Y	Y	Y	NA	Low
Katz et al. (2012) [35]	Case report	Y	Y	Y	Y	NA	NA	NA	Y	-	-	Low
Niu et al. (2016) [36]	Case series	Y	Y	Y	U	Y	Y	Y	Y	Y	Y	Low
Gagliardi et al. (2023) [37]	Case series	Y	Y	Y	Y	Y	Y	Y	Y	Y	NA	Low

Supplementary Table S1. Critical appraisal and risk of bias assessment of the included studies using the Joanna Briggs Institute (JBI) checklists. Overall risk of bias was defined as Low (high quality, ≥ 75% “Yes” scores), Moderate (moderate quality, 50-74% “Yes” scores), or High (low quality, < 50% “Yes” scores). This percentage-based labelling is intended as a descriptive summary of item-level checklist responses rather than a validated risk-of-bias classification, as the JBI checklists do not themselves specify a percentage cut-off for converting checklist scores into risk categories.

Abbreviations: Y = Yes; N = No; U = Unclear; NA = Not Applicable; **-** = Item does not present in the specific checklist.

Note: Q1-Q10 correspond to the specific items of the Joanna Briggs Institute (JBI) Critical Appraisal Checklists. Reference numbers correspond to the bibliography of the main manuscript.
